# Systemic administration of ivabradine, a hyperpolarization‐activated cyclic nucleotide‐gated channel inhibitor, blocks spontaneous absence seizures

**DOI:** 10.1111/epi.16926

**Published:** 2021-05-20

**Authors:** Yasmine Iacone, Tatiana P. Morais, François David, Francis Delicata, Joanna Sandle, Timea Raffai, Harri Rheinallt Parri, Johan Juhl Weisser, Christoffer Bundgaard, Ib Vestergaard Klewe, Gábor Tamás, Morten Skøtt Thomsen, Vincenzo Crunelli, Magor L. Lőrincz

**Affiliations:** ^1^ Neuroscience Research H. Lundbeck A/S, Valby Copenhagen Denmark; ^2^ Biomedical Sciences Faculty of Health and Medical Sciences Copenhagen University Copenhagen Denmark; ^3^ Neuroscience Division School of Biosciences Cardiff University Cardiff UK; ^4^ Integrative Neuroscience and Cognition Center University of Paris Paris France; ^5^ School of Life and Health Sciences Aston University Birmingham UK; ^6^ Department of Anatomy, Physiology, and Neuroscience MTA‐SZTE Research Group for Cortical Microcircuits University of Szeged Szeged Hungary; ^7^ Department of Physiology, Anatomy, and Neuroscience Faculty of Sciences University of Szeged Szeged Hungary; ^8^ Department of Physiology Faculty of Medicine University of Szeged Szeged Hungary; ^9^ Department of Physiology and Biochemistry Faculty of Medicine and Surgery University of Malta Msida Malta

**Keywords:** childhood absence epilepsy, cortex, I_h_ current, thalamocortical neurons, thalamus

## Abstract

**Objective:**

Hyperpolarization‐activated cyclic nucleotide‐gated (HCN) channels are known to be involved in the generation of absence seizures (ASs), and there is evidence that cortical and thalamic HCN channel dysfunctions may have a proabsence role. Many HCN channel blockers are available, but their role in ASs has been investigated only by localized brain injection or in in vitro model systems due to their limited brain availability. Here, we investigated the effect on ASs of orally administered ivabradine (an HCN channel blocker approved for the treatment of heart failure in humans) following injection of the P‐glycoprotein inhibitor elacridar, which is known to increase penetration into the brain of drug substrates for this efflux transporter. The action of ivabradine was also tested following in vivo microinjection into the cortical initiation network (CIN) of the somatosensory cortex and in the thalamic ventrobasal nucleus (VB) as well as on cortical and thalamocortical neurons in brain slices.

**Methods:**

We used electroencephalographic recordings in freely moving Genetic Absence Epilepsy Rats From Strasbourg (GAERSs) to assess the action of oral administration of ivabradine, with and without elacridar, on ASs. Ivabradine was also microinjected into the CIN and VB of GAERSs in vivo and applied to Wistar CIN and GAERS VB slices while recording patch‐clamped cortical Layer 5/6 and thalamocortical neurons, respectively.

**Results:**

Oral administration of ivabradine markedly and dose‐dependently reduced ASs. Ivabradine injection into CIN abolished ASs and elicited small‐amplitude 4–7‐Hz waves (without spikes), whereas in the VB it was less potent. Moreover, ivabradine applied to GAERS VB and Wistar CIN slices selectively decreased HCN channel‐dependent properties of cortical Layer 5/6 pyramidal and thalamocortical neurons, respectively.

**Significance:**

These results provide the first demonstration of the antiabsence action of a systemically administered HCN channel blocker, indicating the potential of this class of drugs as a novel therapeutic avenue for ASs.

**Key Points**

- Systemic administration of ivabradine prevents absence seizures by blocking neuronal HCN channels
- Ivabradine injected in the cortical initiation network abolishes absence seizures, whereas its antiabsence effect is smaller when injected in the ventrobasal thalamus
- HCN channel blockade by ivabradine affects membrane properties of cortical Layer 5/6 pyramidal and thalamocortical neurons

​

​

## 1 INTRODUCTION

Absence seizures (ASs) are characterized by loss of consciousness and lack of voluntary movements accompanied by generalized spike‐and‐wave discharges (SWDs) in the electroencephalogram (EEG). ASs are present in several epilepsies and are the only clinical symptom of childhood absence epilepsy (CAE),^1^, ^2^ which accounts for 10%–17% of all children with epilepsy^3^ and carries a burdensome personal, familial, and societal impact.^4^, ^5^ The first‐line therapy for CAE is ethosuximide monotherapy, followed by valproic acid or lamotrigine,^6^ but about 30% of CAE children are pharmacoresistant, resulting in polytherapy and a consequent marked increase in adverse effects.^7^, ^8^, ^9^ Furthermore, about 60% of children with AS experience neuropsychiatric comorbidities (mainly attention/cognitive impairments), which can be present before the epilepsy diagnosis, and can even be aggravated following full pharmacological control of ASs.^10^, ^11^ Hence, there is a pressing need to find new effective targets for AS treatment.

The role for hyperpolarization‐activated cyclic nucleotide‐gated (HCN) channels in ASs has been extensively investigated, with many studies reporting a gain or loss of function, mainly involving HCN1 and HCN2 subtypes. In particular, analysis of recombinant *HCN2* variants in humans with febrile seizures and genetic epilepsy with febrile seizures plus shows an increased hyperpolarization‐activated current (I_h_), the current generated by HCN channels.^12^ Moreover, several *HCN1* variants have been identified in children with early infantile epileptic encephalopathy that lead to either a gain or a loss of function,^13^ whereas in sporadic idiopathic generalized epilepsy patients, point mutations of *HCN2* give rise to a channel loss of function.^14^ The diversity of these HCN channel dysregulations, however, together with the finding that ASs are not the only phenotype present in these patient cohorts, makes it difficult to draw any firm conclusion on the precise role of HCN channels in human ASs.

Studies in experimental animals have also demonstrated a critical role for HCN channels in ASs, but the results are often contradictory. Global knockout of HCN1^15^ or HCN2^16^ elicits ASs, suggesting an antiabsence role of these channels. Furthermore, two genetic AS models showed both an increased thalamic and a decreased cortical I_h_ as well as a contrasting up‐ or downregulation of HCN1 in both thalamus and cortex,^17^, ^18^, ^19^, ^20^ two brain regions that are essential for AS generation.^21^, ^22^ Moreover, removal of the cyclic adenosine monophosphate (cAMP)‐sensitivity of HCN2 in the whole brain or thalamic ventrobasal nucleus (VB)‐selective HCN2 knockdown leads to ASs,^23^ whereas VB‐selective HCN4 knockdown has no effect.^24^ Conversely, pharmacological and genetic suppression of HCN channels in the VB suppressed ASs in three animal models, suggesting that HCN channels in this brain region have a proabsence role.^25^

Notwithstanding the therapeutic potential of targeting HCN channels for the treatment of ASs, an HCN channel modulator must show efficacy following systemic administration for it to have clinical applicability. Several HCN channel blockers have been developed so far,^26^ including ivabradine (IVA; Procoralan, Corlanor), a drug approved for the treatment of heart failure.^27^, ^28^, ^29^ All these HCN channel blockers, however, show a very limited ability to cross the blood–brain barrier (BBB), due to the efflux mediated by P‐glycoproteins (Pgp). Thus, whereas HCN channel blockers have been extensively used to inhibit neuronal HCN channels in vitro,^30^ the interpretation of the few brain investigations that used systemic administration are questionable due to their poor brain penetration.^31^, ^32^, ^33^

Here, we show for the first time that IVA orally administered together with elacridar (ELA), a Pgp inhibitor,^34^ elicits a marked and long‐lasting reduction of ASs in a well‐established AS model (the Genetic Absence Epilepsy Rats From Strasbourg [GAERSs]).^35^ Additionally, a similar antiabsence action occurs when IVA is directly injected into the cortical initiation network (CIN)^36^ and the VB, and IVA selectively decreases HCN channel‐dependent properties of cortical Layer 5/6 pyramidal and VB thalamocortical neurons in vitro.

## 2 MATERIALS AND METHODS

### 2.1 Animals

GAERSs (originally obtained from Strasbourg, France) were bred and maintained at Cardiff University (UK) and University of Szeged (Hungary). Wistar rats (originally from Envigo) were maintained at H. Lundbeck (Copenhagen, Denmark) and University of Szeged (Szeged, Hungary). Animals were provided with normal diet and water ad libitum, and kept under a light:dark cycle of 12:12 h with light on at 7:00 a.m. Experimental procedures were performed in agreement with the UK Animals (Scientific Procedures) Act (1986), the European Communities Council Directive (2010/63/EU), and the Danish legislation (Law and Order on Animal Experiments; Act No. 474 of May 15, 2014 and Order No. 12 of January 7, 2016).

### 2.2 IVA plasma and brain bioanalysis

Plasma and brain concentrations of IVA were measured in 3‐month‐old male Wistar rats 1 h after injection to assess its concentration at the time of the ELA peak brain concentration, and in 3–4‐month old male GAERSs 2 h after the injection (i.e., at the end of the recording session) to measure brain IVA concentration at the time of the last recording period (see below). Blood samples were kept in 1.6‐mg EDTA/ml of blood, centrifuged at 3300 × *g* for 15 min at 4°C, and stored at −80°C until bioanalysis. Brains were stored at −80°C until bioanalysis.

Brain samples were prepared by homogenizing half brain using isothermal focused acoustic ultrasonication (Covaris E220x) as described previously.^37^ After homogenization, calibration standards and quality control (QC) samples were prepared in blank rat plasma and brain homogenate in the range of .5–500 ng/ml. On the day of analysis, 25 µl of brain homogenates, plasma samples, calibration standards, and QC samples were precipitated with 100 µl acetonitrile containing internal standard. After centrifugation (20 min at 3500 × *g*), 50 µl supernatant was transferred to a 300‐µl 96‐well plate and mixed with 150 µl 25% acetonitrile solution.

IVA concentrations were determined using ultraperformance liquid chromatography (Waters ACQUITY UPLC System) coupled to a tandem mass spectrometry detector (Waters Xevo TQ‐XS). Chromatographic separation was performed on a Waters C18SB HSS column (30 × 2.1 mm, 1.8‐μm particles) with a column compartment temperature of 40°C using gradient elution with mobile phases consisting of .1% formic acid in water and .1% formic acid in acetonitrile. Autosampler temperature was 10°C, and injection volume was 5 µl. Electrospray ionization was performed in positive mode. For IVA, the ion 469.3^+^→262.1^+^ was monitored.

### 2.3 Surgical procedures

Adult male GAERSs (250–300 g) were anesthetized with isoflurane (2%–5%), and body temperature was maintained at 37°C with a heating pad. Six gold‐plated epidural screws (Svenska Dentorama) were implanted in pairs, at frontal, parietal, and cerebellar sites. For microinjection into the VB and CIN, one 8‐mm‐long guide cannula (Bilaney C315G/50‐99) was also implanted in both hemispheres (VB: anteroposterior [AP] −3.2, mediolateral [ML] ±3.6, dorsoventral [DV] 4.5, with a 5° angle; CIN: AP −2.52, ML ±4.8, DV 1.3, [mm from Bregma]).^38^ The animals were allowed to recover for at least 5 days prior to experiments.

### 2.4 EEG recordings

The day before experiments, rats were connected to the recording apparatus and placed individually in a plexiglass box within a Faraday cage for 1–2‐h habituation. On the day of the recordings, animals were placed into the plexiglass box for 30 min (habituation period) followed by 40 min of recording (control period). They were then transferred to an anesthesia induction chamber, slightly anesthetized with isoflurane (1%) and injected intravenously with either ELA (5 mg/kg, 5 ml/kg) or vehicle (VEH1; hydroxypropyl‐β‐cyclodextrin) as soon as the righting reflex was lost. Anesthesia was then terminated, and 20 min after the intravenous ELA (or VEH1) administration, the animals received either IVA (10, 20 or 30 mg/kg) or vehicle (VEH2; 5% D‐glucose in distilled water) orally. Rats were then placed in the plexiglass box, reconnected to the EEG apparatus, and recorded for 2 h while being continuously monitored by one researcher. Drug‐treatments (VEH1‐VEH2, ELA‐VEH2, VEH1‐IVA, and ELA‐IVA) were assigned in a pseudorandom manner with a crossover design. Each animal received a maximum of four different treatments with at least 5 days between each treatment.

For microinjection experiments, on the day of the experiment the animals were recorded for 1 h (control period), followed by the bilateral insertion of a 9‐mm (Bilaney C315I/20‐49) cannula, which was connected to a micropump (Linton Instruments CMA 400). Animals were then injected with either artificial cerebrospinal fluid (aCSF) or IVA (6 nmol) using a flow rate of .25 µl/min for 2 min and recorded for 2 h.

To check cannulae position, brains were collected and washed in phosphate‐buffered saline (10 mmol·L^–1^) followed by fixation in 4% paraformaldehyde for 24 h. After fixation, 100‐µm coronal slices were cut from the region containing the CIN or VB and then mounted with VECTASHIELD Antifade Mounting Media (Vector Laboratories). Slices were imaged within the next 24 h and photographed with a BX61 microscope (Olympus) with a ×4 objective. Results from animals with misplaced cannulae position were not included in the final analysis.

### 2.5 Data acquisition and analysis

#### 2.5.1 SWD detection

The analog EEG signal was acquired through a four‐channel differential preamplifier (high‐pass filter .1 Hz; SuperTech) connected to a four‐channel BioAmp amplifier (1000 gain, low‐pass filter 500 Hz; SuperTech) and digitized at 1000 Hz using a CED Mk3 1401 (Cambridge Electronic Design). SWDs were initially detected semiautomatically using the SeizureDetect script (kindly provided by Steve Clifford, Cambridge Electronic Design) in Spike2 v7.03 (Cambridge Electronic Design), and then checked by visual inspection. Data were digitally processed, and an interictal EEG period of wakefulness was manually selected and used to set a threshold of ±5–8 SD of the baseline EEG. To identify SWDs, all crossings above or below the threshold were then grouped into bursts according to five preset parameters: a maximum onset interval (.2 s), a maximum interval (.75 s), a minimum number of spikes (five), a minimum interval within bursts (1 s), and a minimum duration (.6 s). Identified bursts lasting less than 1 s were discarded. The putative bursts were ultimately classified into SWDs according to the frequency, which was manually set between 5 and 12 Hz to exclude deep sleep epochs or artifacts. This semiautomatic detection was further refined by visual inspection. The following parameters were extracted from the EEG data in 20‐min epochs: the total time spent in seizure, the total number of seizures, and the average duration of a seizure. Treatment data were normalized to the respective control period, and statistical analysis was performed after normalization (see Section 2.6).

#### 2.5.2 Power spectral analysis

The Welch power spectral density analysis was performed with MATLAB (R2019a; MathWorks) on interictal EEG periods that were devoid of ASs in GAERSs treated with oral administration of IVA and other drug combinations. Change of power spectrum density between control and treatment periods was measured as baseline percentage. Statistical analysis was performed after normalization to the respective control period (see Section 2.6). Similar power spectra were performed on the EEG of GAERSs that received IVA directly in the CIN and VB, and compared to VEH injection.

#### 2.5.3 Cortical and thalamic slice preparation, whole‐cell recordings, and data analysis

Male Wistar rats and GAERSs (both 25–35 days old) were anesthetized (ketamine/xylazine, 80/8 mg/kg), their brains were quickly sliced (320 µm thickness) in the coronal plane, and slices containing either the CIN or the VB were incubated at room temperature (20°C) in aCSF containing (in mmol·L^–1^) 130 NaCl, 3.5 KCl, 1 NaH_2_PO_4_, 24 NaHCO_3_, 1 CaCl_2_, 3 MgSO_4_, 10 glucose. For recording, slices were submerged in a chamber perfused with a warmed (35°C) continuously oxygenated (95% O_2_, 5% CO_2_) aCSF containing (in mmol·L^–1^) 130 NaCl, 3.5 KCl, 1 KH_2_PO_4_, 24 NaHCO_3_, 1 MgSO_4_, 2 CaCl_2_, and 10 glucose.

Whole‐cell patch‐clamp recordings were performed using an EPC9 amplifier (Heka Elektronik). Patch pipettes (tip resistance = 4–5 MΩ) were filled with an internal solution containing the following (in mmol·L^–1^): 126 K‐gluconate, 4 KCl, 4 adenosine triphosphate–Mg, .3 guanosine triphosphate–Na_2_, 10 hydroxyethylpiperazine ethane sulfonic acid, 10 creatine phosphate (pH 7.25, osmolarity = 275 mOsm). The liquid junction potential (−13 mV) was corrected offline. Access and series resistances were constantly monitored, and data from neurons with a greater than 20% change from the initial value were discarded. Action potential amplitude was measured from threshold (20 mV/ms on the first derivative of the membrane potential) to the peak of the action potential. Analysis of these whole‐cell data was performed using custom routines written in Igor.

### 2.6 Statistical analysis

Statistical analysis was performed using Prism version 9.0 (GraphPad Software). Normality of the data was verified with a Q‐Q plot. The comparison between the two doses of ELA was performed using an unpaired *t*‐test assuming equal variances between the two groups. The effect of each treatment on SWDs following systemic injection was assessed by repeated‐measurement two‐way analysis of variance (ANOVA) using Sidak correction for multiple comparisons. The main effect of treatment versus vehicle was also measured as area under the curve (AUC) and analyzed with one‐way ANOVA using Dunnett multiple comparisons test. Statistical analysis of the power spectra was carried out with one‐sided Wilcoxon test comparing treatment with control. The main effect of IVA versus aCSF for CIN and VB microinjections was assessed by two‐way ANOVA for multiple timepoints and unpaired *t*‐test for AUC. Data from in vitro recordings in cortical and thalamic neurons were analyzed with Wilcoxon signed rank test.

All quantitative data in the text and figures are reported as mean ± SEM, unless stated otherwise.

### 2.7 Drugs

IVA (3‐[3‐({[(7S)‐3,4‐dimethoxybicyclo[4.2.0]octa‐1,3,5‐trien‐7‐yl]methyl}(methyl) amino) propyl]‐7,8‐dimethoxy‐2,3,4,5‐tetrahydro‐1H‐3‐benzazepin‐2‐one hydrochloride) and ELA (N‐[4‐[2‐(3,4‐dihydro‐6,7‐dimethoxy‐2(1H)‐isoquinolinyl)ethyl]phenyl]‐9,10‐dihydro‐5‐methoxy‐9‐oxo‐4‐acridinecarboxamide) were purchased from Sigma‐Aldrich. For systemic injections, IVA was dissolved in a 5% D‐glucose (Sigma‐Aldrich) solution, and the pH was adjusted to 4. ELA was dissolved in 10% hydroxypropyl‐β‐cyclodextrin, and the pH was adjusted to 4. For local microinjections, IVA was diluted in aCSF. All drugs were freshly prepared on each day of experiments.

## 3 RESULTS

### 3.1 Systemic injection of IVA

**FIGURE 1:**
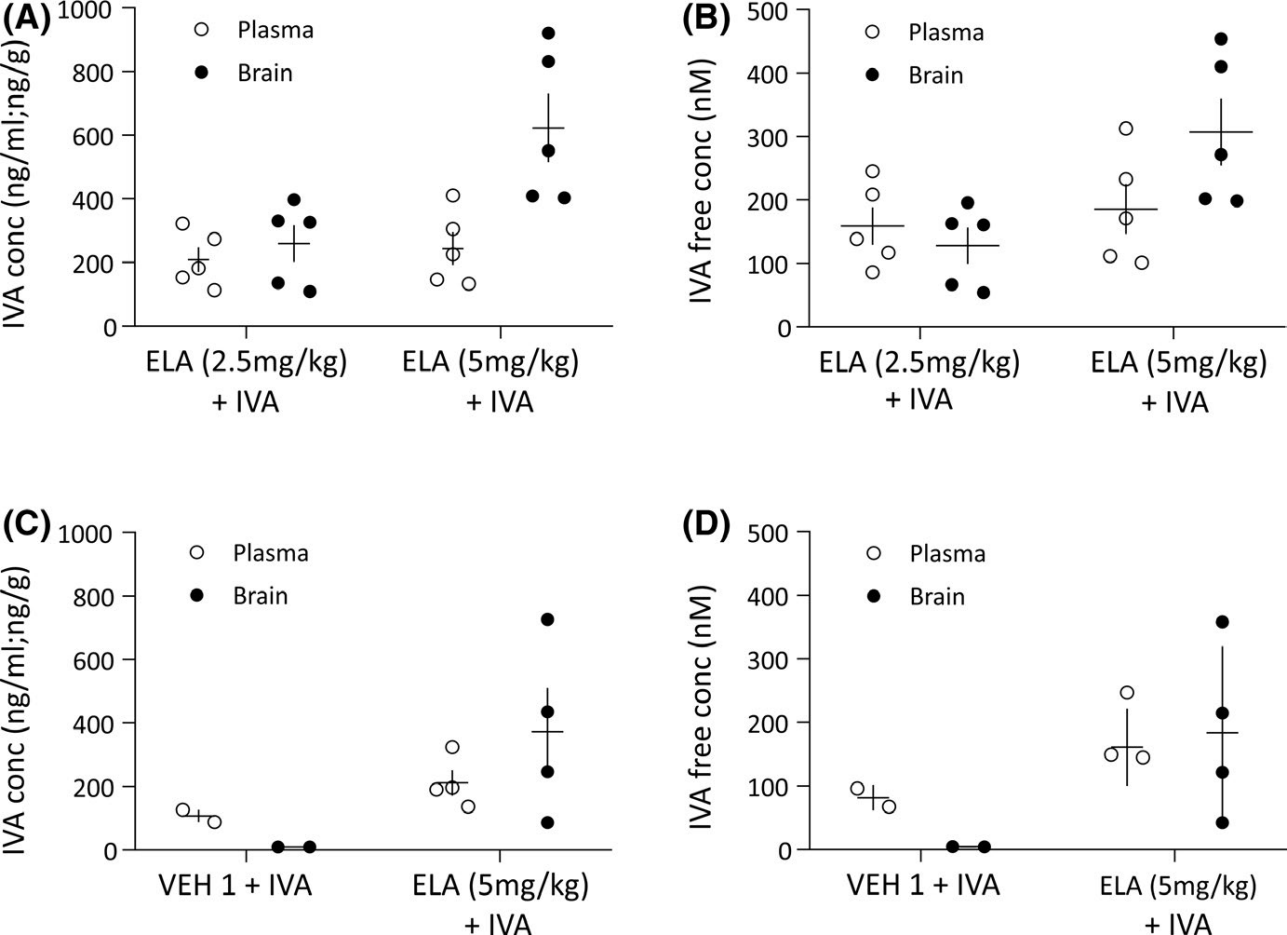
Brain and plasma levels of systemically injected ivabradine (IVA) with and without pretreatment with elacridar. (A, B) Total brain (black dots) and plasma (white dots) levels (A) and brain and plasma free concentrations (B) measured 1 h after oral administration of IVA (30 mg/kg) in Wistar rats (*n* = 5 for each group) that had been pretreated with intravenous injection of either 2.5 or 5 mg/kg elacridar (ELA) 30 min before IVA injection. (C, D) Total brain (black dots) and plasma (white dots) levels (C) and brain and plasma free concentrations (D) of IVA in Genetic Absence Epilepsy Rats From Strasbourg treated with vehicle (VEH) 1 ​+ IVA (*n* = 2) or ELA (5 mg/kg) + IVA (*n* = 4). Animals were sacrificed at the end of the electroencephalographic recordings, that is, 2 h after IVA injection. In A–D, horizontal and vertical lines indicate mean and ±SEM, respectively. conc, concentration

The highest dose of IVA (30 mg/kg) used in this study was selected on the basis of its efficacy and safety as described in previous European Medicines Agency and US Food and Drug Administration reports.^27^, ^28^ For selecting a dose of ELA that could lead to suitable brain levels of IVA, we tested two doses of ELA that had been previously reported to allow good brain penetration of other systemically dosed Pgp substrates.^39^ Intravenous pretreatment of Wistar rats with 5 mg/kg ELA provided higher brain concentration of orally administered IVA (622 ± 107 ng/g,) compared to 2.5 mg/kg ELA (259 ± 57.6 ng/g, *p* = .018; Figure 1A). Similarly, the brain free concentration of IVA was higher in animals pretreated with 5 mg/kg than 2.5 mg/kg ELA (307 ± 53.0 and 128 ± 28.4 nmol·L^–1^, respectively, *p* = .018), showing a direct correlation with the total drug concentration (Figure 1B). No significant differences in the IVA plasma levels were observed in animals pretreated with 2.5 and 5 mg/kg ELA (*p* = .603; Figure 1A,B). A dose of 5 mg/kg ELA was thus used in further experiments.

**FIGURE 2:**
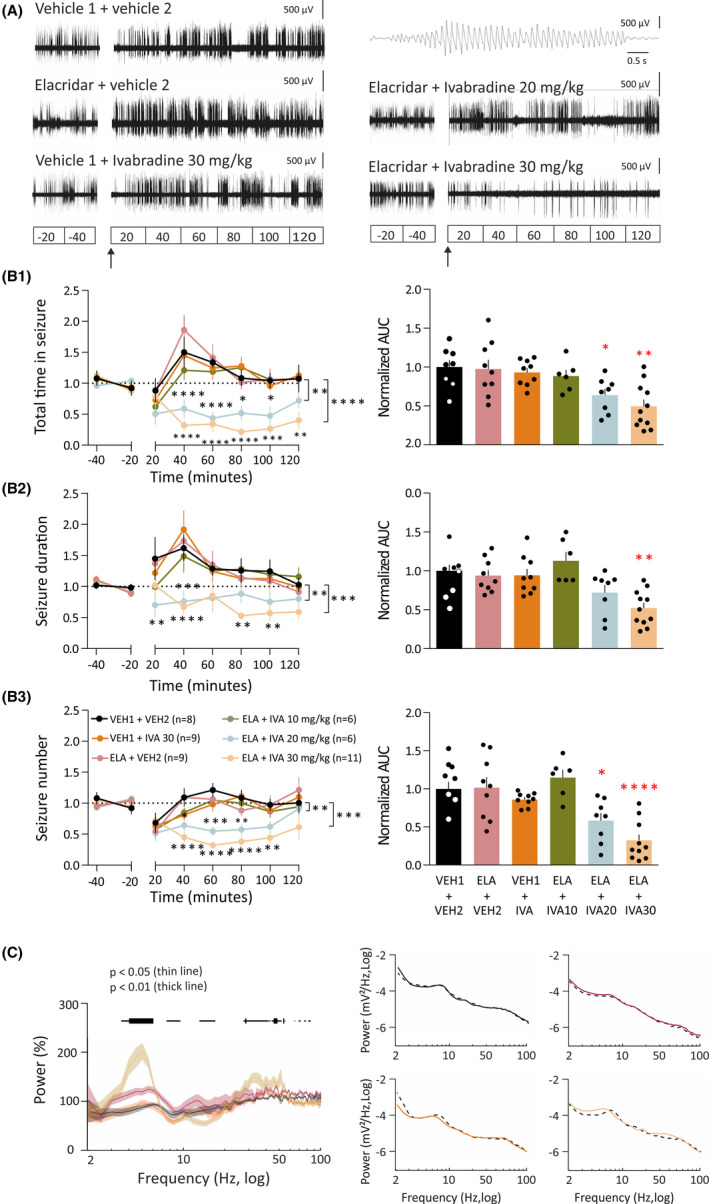
Oral administration of ivabradine (IVA) markedly blocks absence seizures. (A) Representative electroencephalographic (EEG) traces of four different freely moving Genetic Absence Epilepsy Rats From Strasbourg injected with either vehicle (VEH) 1 + VEH2, VEH1 + IVA, elacridar (ELA) + VEH2, ELA + IVA10, ELA + IVA20, or ELA + IVA30. Note the marked reduction in spike‐and‐wave discharges (SWDs) in the ELA + IVA30 group compared to all other groups (a typical SWD is shown enlarged in the top trace on the right). In A and the left plots of B1–3, interruption in the traces is due to the animals being disconnected from the EEG wires for drug administration; ELA (or VEH1) was injected at Time −20 min and IVA (or VEH2) at Time 0 (indicated by the black arrow). Data in B1–3 are normalized to the control period (Section 2). (B1) Time–response curves (left graph) and area under the curve (AUC) of the whole treatment period (right graph) of the total time spent in seizures for VEH1 + VEH2 (black, *n* = 9), ELA + VEH2 (red, *n* = 9), VEH1 + IVA (orange, *n* = 9), ELA + IVA10 (green, *n* = 6), ELA + IVA20 (blue, *n* = 8), and ELA + IVA (ocher, *n* = 11) groups. (B2) Time–response curve (left graph) and AUC (right graph) of the whole treatment period of seizure duration for the six treatment groups (color code and number of animals as in B1). (B3) Time–response curve (left graph) and AUC (right graph) of the whole treatment period of the number of seizures for the six treatment groups (color code and number of animals as in B1). (C) Left graph: Average normalized interictal power spectra of the four treatment groups (number of animals as in B1, except VEH1 + VEH2, *n* = 6). Horizontal bars on top indicate statistical significance (thin line: *p* < .05; thick line: *p* < .01). Right graph: representative examples of interictal power spectra for individual animals of the VEH1 + VEH2 (black), ELA + VEH2 (red), VEH1 + IVA (orange), and ELA + IVA30 (ocher) groups showing both the control period (dashed black line) and the treatment period. *p < .05, **p < .01, ***p < .005, ****p < .001

We next investigated the effect of orally administered IVA on spontaneous ASs in freely moving GAERSs. No gross behavioral changes were observed in any treatment group during the EEG recordings described below. As shown in Figure 2A, in animals pretreated with 5 mg/kg ELA, oral administration of 20 and 30 (but not 10) mg/kg IVA markedly reduced spontaneous ASs, an effect that for the highest dose was visible as early as 20 min after IVA administration and lasted for the entire duration of the recorded period (2 h). Statistical analysis of the normalized AUC of the entire test period showed a significant reduction (62%) of the total time spent in seizures for the ELA + IVA30 group (.38 ± .09, *F* = 8.77, DFn = 5, DFd = 22, *p* = .0044), but not for the ELA + IVA20 (.53 ± .09, *p* = .07), ELA + IVA10 (1.05 ± .09, *p* > .99), ELA + VEH2 (1.2 ± .16, *p* = .61), and VEH1 + IVA groups (1.12 ± .11, *p* = .86) compared to VEH1 + VEH2 (Figure 2B1). The mean duration of the seizures was also significantly decreased (*F* = 6.67, DFn = 5, DFd = 22, 41%) in the ELA + IVA30 group (.59 ± .07, *p* = .008), but not in the ELA + IVA20 (.66 ± .09, *p* = .09), ELA + IVA10 (1.03 ± .1, *p* > .99), ELA + VEH2 (1.06 ± .08, *p* > .99), and VEH1 + IVA groups (1.06 ± .09, *p* = .95) compared to VEH1 + VEH2 (Figure 2B2). Moreover, the number of seizures showed a significant decrease (45%) in rats treated with ELA + IVA30 (.55 ± .09, *F* = 4.35, DFn = 5, DFd = 46, *p* = .01) but not in the ELA + IVA20 (.71 ± .09, *p* = .24), ELA + IVA10 (.99 ± .09, *p* > .99), ELA + VEH2 (1.08 ± .13, *p* = .98), and VEH1 + IVA groups (1.03 ± .06, *p* = .99) compared to VEH1 + VEH2 (Figure 2B3). Finally, power spectra of the interictal EEG showed the administration of ELA + IVA30 to elicit a significant increase in the power of theta (4–8 Hz) and low gamma (30–50 Hz) frequency bands compared to the VEH1 + VEH2 group and a small decrease in the alpha band (8–14 Hz; Figure 2C).

At the end of the last recording session, the brain and plasma of those rats that had received ELA + IVA30 or VEH + IVA30 as their last treatment were collected to determine the IVA plasma and brain levels. As shown in Figure 1C, the total plasma concentrations of IVA measured 2 h after dosing were in the same range in the two treatment groups (VEH1 + IVA30: 107 ± 19 ng/ml; ELA + IVA30 212 ± 39 ng/ml), whereas the brain concentration was substantially higher in the animals that were dosed with ELA‐IVA30 (373 ± 137 ng/g) compared to those with VEH1 + IVA30 (9.5 ± .3 ng/g). The free brain concentration of IVA was 4.7 ± .1 nmol·L^–1^ for VEH1 + IVA30 and 184 ± 67.8 nmol·L^–1^ for the ELA + IVA30 group (Figure 1D).

### 3.2 Local microinjection of IVA

Because ASs are generated by abnormal firing in corticothalamocortical circuits, we next investigated whether the antiabsence effect of systemically administered IVA was mediated by an action on thalamic and/or cortical regions. Thus, we applied IVA by bilateral microinjection into the VB or CIN of freely moving GAERSs.

**FIGURE 3:**
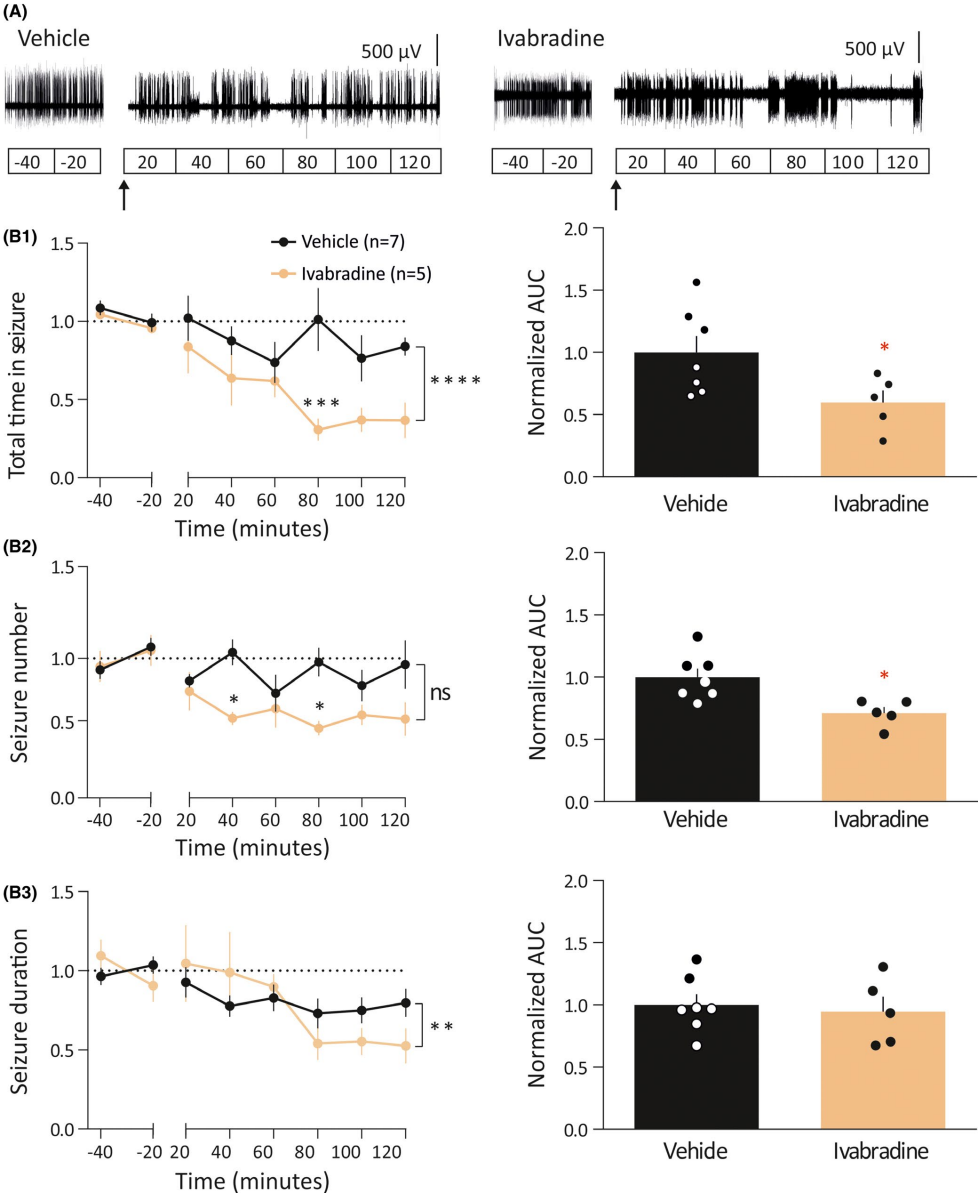
Effect of intrathalamic injection of ivabradine (IVA) on absence seizures. (A) Representative electroencephalographic (EEG) traces of two freely moving Genetic Absence Epilepsy Rats From Strasbourg injected in the ventrobasal nucleus with artificial cerebrospinal fluid (aCSF) or IVA. In A and left graphs of B1–3, the break in the traces indicates the period of time when the EEG wires were disconnected to allow the exchange of the aCSF with the IVA solution in the injection cannula. aCSF and IVA (6 nmol/side) were injected using a flow rate of .25 µl/min for 2 min. In B1–3, data are normalized to the control period. (B1) Time–response curves (left graph) and area under the curve (AUC) of the whole treatment period (right plot) of the total time spent in seizures for aCSF (black, *n* = 7) and IVA (ocher, *n* = 5) injected animals. (B2) Time–response curves (left graph) and AUC of the whole treatment period (right plot) of seizure number of aCSF‐ and IVA‐treated groups (color code and number of animals as in B1). (B3) Time–response curves (left graph) and AUC of the whole treatment period (right plot) of seizure duration for aCSF‐ and IVA‐treated animals (color code and number of animals as in B1). *p < .05, **p < .01, ***p < .005, ****p < .001. ns, not significant

Bilateral microinjection of IVA (6 nmol) into the VB of freely moving GAERSs reduced ASs compared to VEH injection (Figure 3A). Statistical analysis of the AUC of the entire test period showed a significant reduction (40%, *F* = 2.61, DFn = 6, DFd = 4, *p* = .05) of the total time spent in seizures of IVA (.6 ± .09) compared to VEH (Figure 3B1). The number of seizures decreased (29%, *F* = 2.99, DFn = 6, DFd = 4, *p* = .011) in rats treated with IVA (.71 ± .05) compared to VEH (Figure 3B2), but the mean duration of seizures was unchanged (5%, *F* = 1.39, DFn = 6, DFd = 4, *p* = .72; .95 ± .12; Figure 3B3).

**FIGURE 4:**
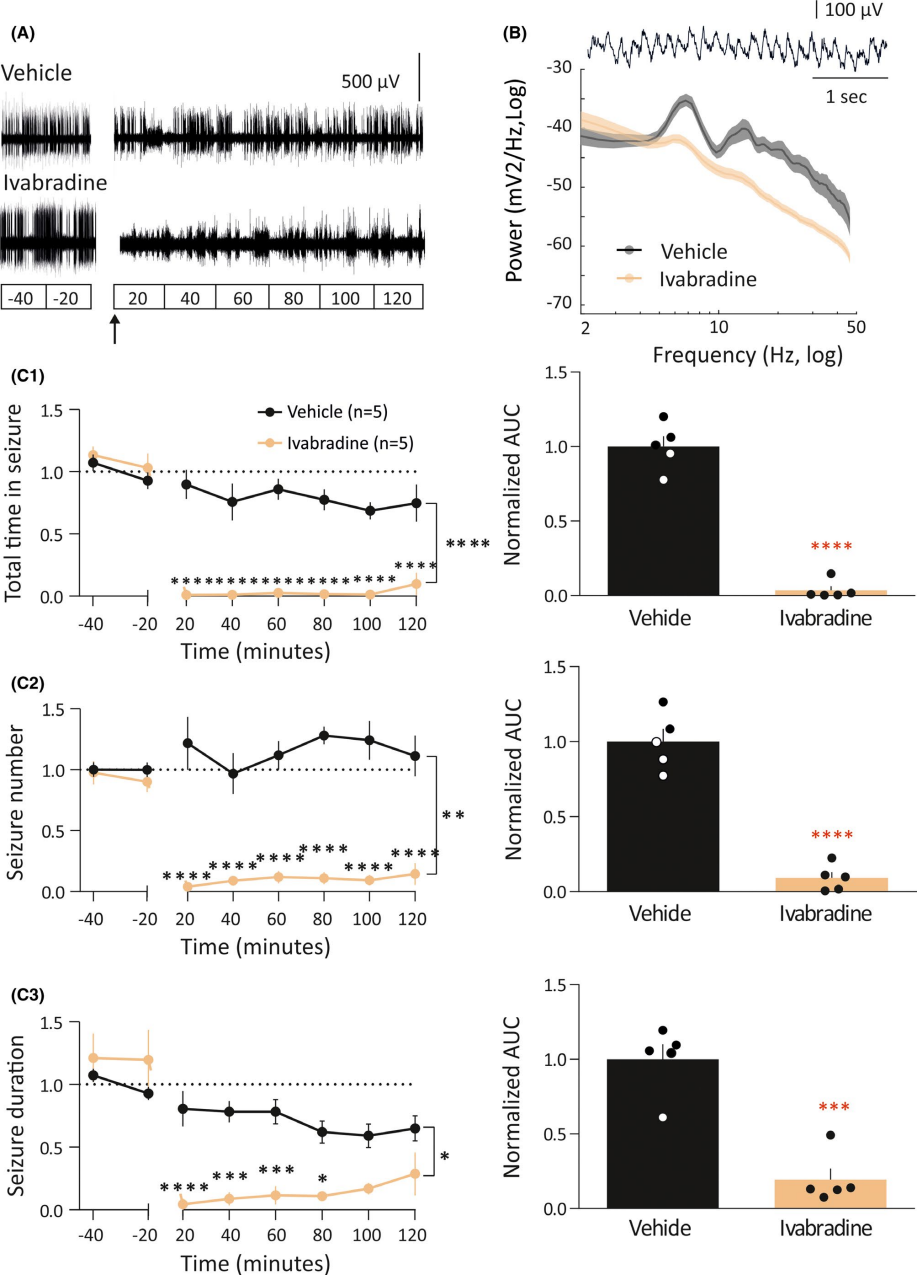
Effect of intra‐cortical initiation network (CIN) injection of ivabradine (IVA) on absence seizures. (A) Representative electroencephalographic (EEG) traces of two freely moving Genetic Absence Epilepsy Rats From Strasbourg injected in the CIN with artificial cerebrospinal fluid (aCSF) or IVA. In A and left graphs of C1–3, the break in the traces indicates the period of time when the EEG wires were disconnected to allow the exchange of the aCSF with the IVA solution in the injection cannula. aCSF and IVA (6 nmol/side) were injected using a flow rate of .25 µl/min for 2 min. In C1–3, data are normalized to the control period. (B) Average power spectra of intra‐CIN injected vehicle and IVA (number of animals and color code as in C1). Example of the 4–7 Hz EEG waveform evoked by IVA is shown on top. (C1) Time–response curves (left graph) and area under the curve (AUC) of the whole treatment period (right plot) of the total time spent in seizures for aCSF (black, *n* = 5) and IVA (ocher, *n* = 5) injected animals. (C2) Time–response curves (left graph) and AUC of the whole treatment period (right plot) of seizure number of aCSF‐ and IVA‐treated groups (color code and number of animals as in C1). (C3) Time–response curves (left graph) and AUC of the whole treatment period (right plot) of seizure duration for aCSF‐ and IVA‐treated animals (color code and number of animals as in C1). *p < .05, **p < .01, ***p < .005, ****p < .001

Microinjection of IVA (6 nmol) in the CIN abolished ASs even in the first 20‐min period after microinjection, and the effect lasted for the entire 2‐h posttreatment recording period (Figure 4A,C1). The AUC of the total time spent in seizures after IVA (.04 ± .03) microinjection was 96% (*F* = 6.23, DFn = 4, DFd = 4, *p* < .0001) smaller than that of VEH (Figure 4C1). Notably, the CIN injection of IVA elicited small‐amplitude waves (with no spikes) at 4–7 Hz (Figure 4A,B); these EEG oscillations were not the electrographic expression of Ass, because they were not accompanied by motor arrest and the rats kept moving around the cage during this EEG activity. The number of seizures showed a 91% (*F* = 4.57, DFn = 4, DFd = 4, *p* < .0001) decrease in rats treated with IVA (.09 ± .04% compared to VEH; Figure 4C2). Likewise, the mean duration of the seizures was also decreased by 81% (*F* = 1.79, DFn = 4, DFd = 4, *p* = .0002) after IVA administration (.19% ± .08% compared to VEH; Figure 4C3).

### 3.3 Effect of IVA on cortical and thalamic neuron properties

**FIGURE 5:**
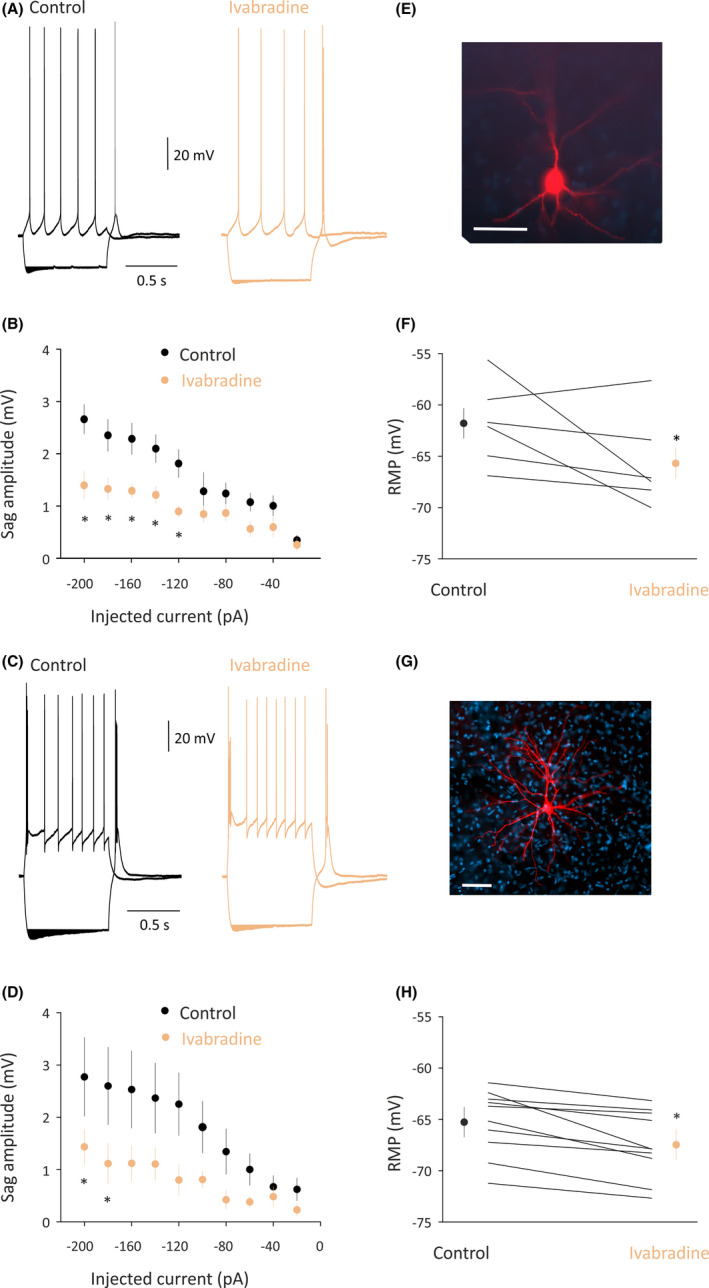
Effect of ivabradine (IVA) on the membrane properties of cortical initiation network (CIN) Layer 5/6 pyramidal and ventrobasal nucleus (VB) thalamocortical neurons in vitro. (A) Representative voltage responses of a Wistar CIN Layer 5 pyramidal neuron to a hyperpolarizing and depolarizing current step (−200 and 80 pA, respectively) before (control) and during application of 30 µmol·L^–1^ IVA (membrane potential = −58 mV). Note the decreased depolarizing sag in the presence of IVA. (B) Photomicrograph of the cortical Layer 5 neuron from which the electrical recordings shown in A were made. Scale bar = 50 µm. (C) Plot of sag amplitude versus injected current show a decrease of the sag in the presence of IVA that is significant for the largest injected currents. (D) Resting membrane potential (RMP) in control conditions and in the presence of IVA. Large symbols indicate mean ± SEM. (E) Representative voltage responses of a Genetic Absence Epilepsy Rat From Strasbourg VB thalamocortical neuron to a hyperpolarizing and depolarizing current step (−200 and 100 pA, respectively) before (control) and during application of 30 µmol·L^–1^ IVA (membrane potential = −61 mV). Note the decreased depolarizing sag in the presence of IVA. (F) Photomicrograph of the thalamocortical neuron from which the electrical recordings shown in E were made. Scale bar = 50 µm. (G) Plot of sag amplitude versus injected current shows a decrease of the sag in the presence of IVA that is significant for the largest injected currents (*p* < .05). (H) RMP in control conditions and in the presence of IVA. Large symbols indicate mean ± SEM. *p < .05

Because the cellular effects of IVA in neurons of key brain areas for AS generation have not been studied either in normal nonepileptic animals or in GAERSs, we investigated the ability of this drug to block HCN channel‐mediated membrane properties of GAERS VB thalamocortical neurons (Figure 5F). To exclude the possibility that any effect of IVA might be due to the higher I_h_ current present in GAERSs, we also studied the action of IVA in cortical Layer 5/6 pyramidal neurons of normal Wistar rats (Figure 5B). IVA (3 ​µmol·L^–1^) blocked the characteristic depolarizing sag elicited in these neurons by hyperpolarizing current pulses (Figure 5A,C,E,G). Furthermore, IVA hyperpolarized the membrane potential of both neuronal types (Figure 5D,H) and decreased the number of action potentials evoked by a low‐threshold spike in thalamocortical and cortical neurons (VB: control 5.3 ± .8, IVA 4.5 ± .9, *n* = 10, *p* < .05; CIN: control 2.5 ± .28, IVA 1.0 ± .4, *n* = 6, *p* < .05). In contrast, IVA had no effect on the number of action potentials evoked by depolarizing current pulses (VB: control 7.9 ± .4, IVA 7.6 ± .6, *n* = 10, *p* > .05; CIN: control 4.6 ± .2, IVA 4.5 ± .2, *n* = 6, *p* > .05), and the action potential amplitude (VB: control 71.2 ± 6.8 mV, IVA 71.4 ± 6.5 mV, *n* = 11, *p* > .05; CIN: control 78.05 ± 4.75 mV, IVA 80.51 ± 5.32 mV, *n* = 6, *p* > .05) and threshold (VB: control −51.3 ± 3.3 mV, IVA −51.7 ± 2.7 mV, *n* = 11, *p* > .05; CIN: control −43.7 ± 2.3 mV, IVA −42.6 ± 2.2 mV, *n* = 6, *p* > .05), indicating that the effect of this drug is selective on HCN channel‐mediated membrane properties in both Wistar CIN cortical Layer 5/6 pyramidal and GAERS VB thalamocortical neurons.

## 4 DISCUSSION

Our study provides the first demonstration of the potent antiabsence action of systemic administration of the HCN channel blocker IVA. Moreover, this drug abolishes and reduces ASs when microinjected directly into the CIN and VB, respectively, actions mediated by its ability to decrease I_h_‐dependent properties of CIN Layer 5/6 pyramidal and VB thalamocortical neurons in vitro.

Targeting brain HCN channels in vivo has been a major challenge due to the inability of available HCN channel‐acting drugs to cross the BBB and provide long‐lasting central nervous system effects. Here, by inhibiting Pgp with ELA,^34^, ^39^, ^40^ we achieved sufficient IVA brain concentrations even 2 h after oral administration to affect physiological brain rhythms, namely, interictal alpha, theta, and gamma waves, and pathological activity, namely, ASs. In the absence of ELA pretreatment, IVA failed to alter normal and paroxysmal brain oscillations. Notably, the block of Pgp by ELA also allowed for the fast antiabsence action of orally administered IVA observed in this study. In contrast, IVA decreased pharmacologically and electrically induced convulsive seizures without pretreatment with a drug capable of improving its brain absorption.^31^, ^32^, ^33^ IVA brain and plasma levels were not measured in these studies, and given our results and previous evidence of IVA inability to substantially penetrate the brain,^27^, ^28^, ^29^, ^30^ it is at present difficult to explain the IVA anticonvulsant action reported in the above studies.

The antiabsence effect of IVA injected into the VB and the CIN confirms previous results of the critical role of thalamic and cortical HCN channels in ASs.^17^, ^18^, ^19^, ^20^, ^25^ The effect of IVA injected into the CIN is markedly stronger than that following oral administration, whereas IVA injection into the VB shows a small reduction. In contrast, microdialysis injection into the VB of ZD7288, another HCN channel blocker, has a strong antiabsence action.^25^ Although differences in drug potency may explain this difference, a much larger portion of the VB is undoubtedly affected by continuous 2‐h ZD7288 microdialysis^25^, ^41^ compared to the more localized 2‐min IVA microinjected bolus used in the present study.

Our investigation also provides the first in vivo evidence that whole‐brain block of HCN channels affects normal brain oscillations, specifically the interictal increase in theta and gamma band power and a shift of the peak of the theta frequency band. Theta and gamma oscillations have been previously linked to I_h_ and ASs^42^, ^43^, and broad changes in corticothalamocortical firing dynamics induced by blocking I_h_ might underlie both the AS block and changes in interictal oscillations. Moreover, IVA injected into the CIN elicits small‐amplitude waves (with no spikes) at theta frequency (4–7 Hz). The mechanism and pathophysiological significance of all waves induced by systemic and intra‐CIN injection of IVA remain to be established.

IVA modulates I_h_‐dependent membrane properties of Wistar cortical Layer 5/6 pyramidal neurons in the CIN and GAERS thalamocortical neurons in the VB, namely, the depolarizing sag and the resting membrane potential, leaving other membrane properties unaffected, namely, action potential threshold and amplitude. As tonic firing was not affected by IVA, the decrease of action potential number evoked by low‐threshold spikes of thalamocortical neurons can be explained by the smaller I_h_ tail current in the presence of IVA providing a smaller depolarizing contribution to low‐threshold spikes, which in turn generate fewer action potentials.

Our study represents the first proof of principle that whole‐brain pharmacological block of HCN channels has an antiabsence action, and demonstrates for the first time that reduction of HCN function selectively in the CIN abolishes ASs, that is, HCN channels of the CIN are necessary for AS generation. HCN1 channels are more abundantly expressed in cortex than thalamus, whereas HCN2 and HCN4 predominate in thalamocortical neurons.^24^, ^44^ Thus, although IVA is a nonselective inhibitor of HCN channel isoforms, it is likely that HCN1 may be the isoform underlying its action in the CIN, whereas its VB effects may involve an interplay between HCN2 and HCN4. Notably, in normal nonepileptic animals, VB‐selective knockdown of HCN4 does not elicit ASs, whereas VB‐selective HCN2 knockdown, as well as global HCN2^16^ or HCN1^15^ knockout (KO), lead to ASs, suggesting an antiabsence role of both isoforms. In contrast, our previous study^25^ and the present investigation demonstrate that a genetic and pharmacological block of HCN channels in the VB of different mouse and rat models decreases ASs. Possible explanations of these contradictory results include compensatory changes in the full HCN1 and HCN2 KO mice, different species used, different potency of HCN blockers against HCN channel subtypes, and diverse efficacy/selectivity of genetic versus pharmacological means of manipulating HCN channels. The alternative interpretation we suggest here is that (1) in normal animals, thalamic HCN2 has an antiabsence effect^23^ and thalamic HCN4 has a proabsence action^24^, ^45^; and (2) in epileptic animals, there is an increased contribution of HCN4, with respect to HCN2, to the total I_h_ of thalamocortical neurons. Under this hypothesis, in nonepileptic animals, VB‐selective knockdown of HCN2 elicits ASs and VB‐selective knockdown of HCN4 does not.^23^, ^24^ In genetic AS models, a pharmacological or genetic block of all subtypes of thalamic HCN channels would lead to an antiabsence effect.^25^ In support of our hypothesis, (1) in thalamic slices of HCN4 KO mice, there is a reduction in electrically evoked intrathalamic oscillations (which are considered a proxy of thalamic rhythmic paroxysmal activity)^24^; and (2) an increased I_h_ is present in VB thalamocortical neurons of GAERSs^20^ and of normal mice that develop atypical ASs following a cortical infarct.^45^ This increased I_h_ may result from enhanced HCN channel expression,^17^ or from changes in the modulation of this current by intracellular messengers (e.g., cAMP)^46^ and neurotransmitters (e.g., noradrenaline).^47^ Notably, our hypothesis makes the testable predictions that in AS models, a selective block of thalamic HCN4 and HCN2 channels should have an antiabsence and no effect on ASs, respectively. The recent development of a preferential HCN4 blocker makes it now feasible to test this hypothesis.^48^

In conclusion, acute systemic administration, and local cortical and thalamic injection of the HCN channel blocker IVA abolishes genetically determined ASs in a well‐established rat model and modulates various interictal thalamocortical rhythms by acting on thalamic and cortical I_h_‐dependent membrane properties. Whereas the antiabsence role of another I_h_ blocker (ZD788) has been demonstrated in GAERSs, Stargazer mice, and the γ‐hydroxybutyrate model,^41^ the translational significance of the present results still needs to be fully investigated. In particular, it remains to be established whether a similar effect of IVA as shown here in GAERSs is also observed in other well‐established inbred rat models,^49^ monogenic mouse models,^21^ and pharmacological models.^50^ It will also be interesting to investigate the effect of IVA on SWDs and associated motor arrest (i.e., ASs) that are present in various outbred rat strains, although these EEG and behavioral abnormalities have been suggested to represent a physiological trait,^51^ a hypothesis strongly rebutted.^52^, ^53^, ^54^ Moreover, whereas the effects of IVA on various thalamocortical rhythms reported in the present work may restrict its clinical potential, selective blockers of HCN channel isoforms, which may not affect physiological thalamocortical oscillations, could represent lead compounds for future antiabsence drugs.

## CONFLICT OF INTEREST

Y.I., J.J.W., C.B., M.S.T. and I.V.K. are Lundbeck employees. None of the other authors has any conflict of interest to disclose.

## AUTHOR CONTRIBUTIONS

Y.I., T.P.M., M.S.T., I.V.K., G.T., V.C., and M.L.L. designed research. Y.I., T.P.M., F.Da., F.De., J.S., T.R., H.R.P., J.J.W., C.B., and M.L.L. performed research and/or analyzed data. Y.I. wrote the manuscript. M.S.T., V.C., and M.L.L. contributed to critical manuscript revisions; the rest of the authors contributed to final manuscript revisions.

## References

[1] Fisher RS , Cross JH , D'Souza C , French JA , Haut SR , Higurashi N , et al. Instruction manual for the ILAE 2017 operational classification of seizure types. Epilepsia. 2017;58(4):531–42. 28276064 10.1111/epi.13671

[2] Fisher RS , Cross JH , French JA , Higurashi N , Hirsch E , Jansen FE , et al. Operational classification of seizure types by the International League Against Epilepsy: position paper of the ILAE Commission for Classification and Terminology. Epilepsia. 2017;58(4):522–30. 28276060 10.1111/epi.13670

[3] Berg AT , Shinnar S , Levy SR , Testa FM , Smith‐Rapaport S , Beckerman B . How well can epilepsy syndromes be identified at diagnosis? A reassessment 2 years after initial diagnosis. Epilepsia. 2000;41(10):1269–75. 11051121 10.1111/j.1528-1157.2000.tb04604.x

[4] Vega C , Guo J , Killory B , Danielson N , Vestal M , Berman R , et al. Symptoms of anxiety and depression in childhood absence epilepsy. Epilepsia. 2011;52(8):e70–4. 21635244 10.1111/j.1528-1167.2011.03119.x PMC3145036

[5] Nickels K . Seizure and psychosocial outcomes of childhood and juvenile onset generalized epilepsies: wolf in sheep's clothing, or well‐dressed wolf? Epilepsy Curr. 2015;15(3):114–7. 26316843 10.5698/1535-7597-15.3.114 PMC4527134

[6] Glauser TA , Cnaan A , Shinnar S , Hirtz DG , Dlugos D , Masur D , et al. Ethosuximide, valproic acid, and lamotrigine in childhood absence epilepsy. N Engl J Med. 2010;362(9):790–9. 20200383 10.1056/NEJMoa0902014 PMC2924476

[7] Glauser TA , Cnaan A , Shinnar S , Hirtz DG , Dlugos D , Masur D , et al. Ethosuximide, valproic acid, and lamotrigine in childhood absence epilepsy: initial monotherapy outcomes at 12 months. Epilepsia. 2013;54(1):141–55. 23167925 10.1111/epi.12028 PMC3538883

[8] Cnaan A , Shinnar S , Arya R , Adamson PC , Clark PO , Dlugos D , et al. Second monotherapy in childhood absence epilepsy. Neurology. 2017;88(2):182–90. 27986874 10.1212/WNL.0000000000003480 PMC5224720

[9] Kessler SK , McGinnis E . A practical guide to treatment of childhood absence epilepsy. Pediatr Drugs. 2019;21(1):15–24. 10.1007/s40272-019-00325-x PMC6394437 30734897

[10] Shinnar RC , Shinnar S , Cnaan A , Clark P , Dlugos D , Hirtz DG , et al. Pretreatment behavior and subsequent medication effects in childhood absence epilepsy. Neurology. 2017;89(16):1698–706. 28916534 10.1212/WNL.0000000000004514 PMC5644466

[11] Masur D , Shinnar S , Cnaan A , Shinnar RC , Clark P , Wang J , et al. Pretreatment cognitive deficits and treatment effects on attention in childhood absence epilepsy. Neurology. 2013;81(18):1572–80. 24089388 10.1212/WNL.0b013e3182a9f3ca PMC3806916

[12] Dibbens LM , Reid CA , Hodgson B , Thomas EA , Phillips AM , Gazina E , et al. Augmented currents of an HCN2 variant in patients with febrile seizure syndromes. Ann Neurol. 2010;67(4):542–6. 20437590 10.1002/ana.21909 PMC3383007

[13] Nava C , Dalle C , Rastetter A , Striano P , de Kovel CGF , Nabbout R , et al. De novo mutations in HCN1 cause early infantile epileptic encephalopathy. Nat Genet. 2014;46(6):640–5. 24747641 10.1038/ng.2952

[14] DiFrancesco JC , Barbuti A , Milanesi R , Coco S , Bucchi A , Bottelli G , et al. Recessive loss‐of‐function mutation in the pacemaker HCN2 channel causing increased neuronal excitability in a patient with idiopathic generalized epilepsy. J Neurosci. 2011;31(48):17327–37. 22131395 10.1523/JNEUROSCI.3727-11.2011 PMC6623833

[15] Nishitani A , Kunisawa N , Sugimura T , Sato K , Yoshida Y , Suzuki T , et al. Loss of HCN1 subunits causes absence epilepsy in rats. Brain Res. 2018;2019(1706):209–17. 10.1016/j.brainres.2018.11.004 30408474

[16] Ludwig A . Absence epilepsy and sinus dysrhythmia in mice lacking the pacemaker channel HCN2. EMBO J. 2003;22(2):216–24. 12514127 10.1093/emboj/cdg032 PMC140107

[17] Budde T . Impaired regulation of thalamic pacemaker channels through an imbalance of subunit expression in absence epilepsy. J Neurosci. 2005;25(43):9871–82. 16251434 10.1523/JNEUROSCI.2590-05.2005 PMC6725576

[18] Kuisle M , Wanaverbecq N , Brewster AL , Frère SGA , Pinault D , Baram TZ , et al. Functional stabilization of weakened thalamic pacemaker channel regulation in rat absence epilepsy. J Physiol. 2006;575(1):83–100. 16728450 10.1113/jphysiol.2006.110486 PMC1819420

[19] Kole MHP , Bräuer AU , Stuart GJ . Inherited cortical HCN1 channel loss amplifies dendritic calcium electrogenesis and burst firing in a rat absence epilepsy model. J Physiol. 2007;578(2):507–25. 17095562 10.1113/jphysiol.2006.122028 PMC2075144

[20] Cain SM , Tyson JR , Jones KL , Snutch TP . Thalamocortical neurons display suppressed burst‐firing due to an enhanced Ih current in a genetic model of absence epilepsy. Pflugers Arch. 2015;467(6):1367–82. 24953239 10.1007/s00424-014-1549-4 PMC4435665

[21] Crunelli V , Leresche N . Childhood absence epilepsy: genes, channels, neurons and networks. Nat Rev Neurosci. 2002;3(5):371–82. 11988776 10.1038/nrn811

[22] Blumenfeld H . Cellular and network mechanisms of spike‐wave seizures. Epilepsia. 2005;46(S9):21–33. 10.1111/j.1528-1167.2005.00311.x 16302873

[23] Hammelmann V , Stieglitz MS , Hülle H , Le Meur K , Kass J , Brümmer M , et al. Abolishing cAMP sensitivity in HCN2 pacemaker channels induces generalized seizures. JCI Insight. 2019;4(9):e126418. 10.1172/jci.insight.126418 PMC6538325 31045576

[24] Zobeiri M , Chaudhary R , Blaich A , Rottmann M , Herrmann S , Meuth P , et al. The hyperpolarization‐activated HCN4 channel is important for proper maintenance of oscillatory activity in the thalamocortical system. Cereb Cortex. 2019;29(5):2291–304. 30877792 10.1093/cercor/bhz047 PMC6458902

[25] David F , Çarçak N , Furdan S , Onat F , Gould T , Mészáros Á , et al. Suppression of hyperpolarization‐activated cyclic nucleotide‐gated channel function in thalamocortical neurons prevents genetically determined and pharmacologically induced absence seizures. J Neurosci. 2018;38(30):6615–27. 29925625 10.1523/JNEUROSCI.0896-17.2018 PMC6067077

[26] Postea O , Biel M . Exploring HCN channels as novel drug targets. Nat Rev Drug Discov. 2011;10(12):903–14. 22094868 10.1038/nrd3576

[27] US Food and Drug Administration . Corlanor (ivabradine). 2015. Available at: https://www.accessdata.fda.gov/drugsatfda_docs/nda/2015/206143Orig1s000TOC.cfm. Accessed 20 November 2015

[28] European Medicinal Agency . Procoralan (ivabradine). 2005. Available at: https://www.ema.europa.eu/en/medicines/human/EPAR/procoralan. Accessed 29 March 2019

[29] Savelieva I , Camm AJ . Novel I_f_ current inhibitor ivabradine: safety considerations. In: Camm AJ , Tendera M , editors. Heart rate slowing by If current inhibition. Basel, Switzerland: Karger; 2006. p. 79–96. 10.1159/000095430 16936474

[30] Santoro B , Shah MM . Hyperpolarization‐activated cyclic nucleotide‐gated channels as drug targets for neurological disorders. Annu Rev Pharmacol Toxicol. 2020;60(1):109–31. 31914897 10.1146/annurev-pharmtox-010919-023356

[31] Łuszczki JJ , Prystupa A , Andres‐Mach M , Marzęda E , Florek‐Łuszczki M . Ivabradine (a hyperpolarization activated cyclic nucleotide‐gated channel blocker) elevates the threshold for maximal electroshock‐induced tonic seizures in mice. Pharmacol Rep. 2013;65(5):1407–14. 24399738 10.1016/s1734-1140(13)71500-7

[32] Sawicka KM , Wawryniuk A , Zwolak A , Daniluk J , Szpringer M , Florek‐Luszczki M , et al. Influence of ivabradine on the anticonvulsant action of four classical antiepileptic drugs against maximal electroshock‐induced seizures in mice. Neurochem Res. 2017;42(4):1038–43. 28083847 10.1007/s11064-016-2136-1 PMC5375969

[33] Cavalcante TMB , De Melo JMA , Lopes LB , Bessa MC , Santos JG , Vasconcelos LC , et al. Ivabradine possesses anticonvulsant and neuroprotective action in mice. Biomed Pharmacother. 2019;109:2499–512. 30551511 10.1016/j.biopha.2018.11.096

[34] Dash RP , Jayachandra Babu R , Srinivas NR . Therapeutic potential and utility of elacridar with respect to P‐glycoprotein inhibition: an insight from the published in vitro, preclinical and clinical studies. Eur J Drug Metab Pharmacokinet. 2017;42(6):915–33. 28374336 10.1007/s13318-017-0411-4

[35] Depaulis A , David O , Charpier S . The genetic absence epilepsy rat from Strasbourg as a model to decipher the neuronal and network mechanisms of generalized idiopathic epilepsies. J Neurosci Methods. 2016;260:159–74. 26068173 10.1016/j.jneumeth.2015.05.022

[36] Meeren HKM , Pijn JPM , Van Luijtelaar ELJM , Coenen AML , da Lopes Silva FH . Cortical focus drives widespread corticothalamic networks during spontaneous absence seizures in rats. J Neurosci. 2002;22(4):1480–95. 11850474 10.1523/JNEUROSCI.22-04-01480.2002 PMC6757554

[37] Bundgaard C , Eneberg E , Sánchez C . P‐glycoprotein differentially affects escitalopram, levomilnacipran, vilazodone and vortioxetine transport at the mouse blood‐brain barrier in vivo. Neuropharmacology. 2016;103:104–11. 26700248 10.1016/j.neuropharm.2015.12.009

[38] Paxinos G , Watson C . The rat brain. 4th ed. London, UK: Academic Press; 1998.

[39] Kallem R , Kulkarni CP , Patel D , Thakur M , Sinz M , Singh SP , et al. A simplified protocol employing elacridar in rodents: a screening model in drug discovery to assess P‐gp mediated efflux at the blood brain barrier. Drug Metab Lett. 2012;6(2):134–44. 23061481

[40] Davis TP , Sanchez‐Covarubias L , Tome ME . P‐glycoprotein trafficking as a therapeutic target to optimize CNS drug delivery. Adv Pharmacol. 2014;71:25–44. 25307213 10.1016/bs.apha.2014.06.009 PMC4301584

[41] David F , Schmiedt JT , Taylor HL , Orban G , Di Giovanni G , Uebele VN , et al. Essential thalamic contribution to slow waves of natural sleep. J Neurosci. 2013;33(50):19599–610. 24336724 10.1523/JNEUROSCI.3169-13.2013 PMC3858629

[42] Maheshwari A , Marks RL , Yu KM , Noebels JL . Shift in interictal relative gamma power as a novel biomarker for drug response in two mouse models of absence epilepsy. Epilepsia. 2016;57(1):79–88. 26663261 10.1111/epi.13265 PMC5551895

[43] Kocsis B , Li S . In vivo contribution of h‐channels in the septal pacemaker to theta rhythm generation. Eur J Neurosci. 2004;20(8):2149–58. 15450094 10.1111/j.1460-9568.2004.03678.x

[44] Notomi T , Shigemoto R . Immunohistochemical localization of Ih channel subunits, HCN1‐4, in the rat brain. J Comp Neurol. 2004;471(3):241–76. 14991560 10.1002/cne.11039

[45] Paz JT , Davidson TJ , Frechette ES , Delord B , Parada I , Peng K , et al. Closed‐loop optogenetic control of thalamus as a tool for interrupting seizures after cortical injury. Nat Neurosci. 2013;16(1):64–70. 23143518 10.1038/nn.3269 PMC3700812

[46] Pedarzani P , Storm JF . Protein kinase A‐independent modulation of ion channels in the brain by cyclic AMP. Proc Natl Acad Sci U S A. 1995;92(25):11716–20. 8524835 10.1073/pnas.92.25.11716 PMC40473

[47] Pape H‐C , McCormick DA . Noradrenaline and serotonin selectively modulate thalamic burst firing by enhancing a hyperpolarization‐activated cation current. Nature. 1989;340(6236):715–8. 2475782 10.1038/340715a0

[48] Romanelli MN , Del Lungo M , Guandalini L , Zobeiri M , Gyokeres A , Arpadffy‐Lovas T , et al. EC18 as a tool to understand the role of HCN4 channels in mediating hyperpolarization‐activated current in tissues. ACS Med Chem Lett. 2019;10:584–9. 30996800 10.1021/acsmedchemlett.8b00587 PMC6466822

[49] Jarre G , Guillemain I , Deransart C , Depaulis A . Genetic models of absence epilepsy in the rat. In: Pitkänen A , Schwartkroin PA , Galanopoulou AS , Moshé SL , eds. Models of seizures and epilepsy. 2nd ed. London, UK: Academic Press; 2017. p. 455–71.

[50] Venzi M , Di Giovanni G , Crunelli V . A critical evaluation of the gamma‐hydroxybutyrate (GHB) model of absence seizures. CNS Neurosci Ther. 2015;21(2):123–40. 25403866 10.1111/cns.12337 PMC4335601

[51] Taylor JA , Rodgers KM , Bercum FM , Booth CJ , Dudek FE , Barth DS . Voluntary control of epileptiform spike‐wave discharges in awake rats. J Neurosci. 2017;37(24):5861–9. 28522734 10.1523/JNEUROSCI.3235-16.2017 PMC6596506

[52] Taylor JA, Rodgers KM, Bercum FM, Booth CJ, Dudek FE, Barth DS. Voluntary control of epileptiform spike‐wave discharges in awake rats. J Neurosci. 2017;37:5861–9. 28522734 10.1523/JNEUROSCI.3235-16.2017 PMC6596506

[53] Crunelli V , Lőrincz ML , McCafferty C , Lambert RC , Leresche N , Di Giovanni G , et al. Clinical and experimental insight into pathophysiology, comorbidity and therapy of absence seizures. Brain. 2020;143(8):2341–68. 32437558 10.1093/brain/awaa072 PMC7447525

[54] McCafferty C , Gruenbaum B , Tung R, Li J‐J, Salvino P, Vincent P, et al. Decreased overall neuronal activity in a rodent model of impaired consciousness during absence seizures. BioRxiv. 2021. Available at: https://www.biorxiv.org/content/ 10.1101/2021.04.20.440390v1; Accessed 21 April 2021.

